# Perceptual teleology: expectations of action efficiency bias social perception

**DOI:** 10.1098/rspb.2018.0638

**Published:** 2018-08-08

**Authors:** Matthew Hudson, Katrina L. McDonough, Rhys Edwards, Patric Bach

**Affiliations:** School of Psychology, University of Plymouth, Plymouth, UK

**Keywords:** representational momentum, action prediction, prediction errors, teleological reasoning, motion perception, social perception

## Abstract

Primates interpret conspecific behaviour as goal-directed and expect others to achieve goals by the most efficient means possible. While this teleological stance is prominent in evolutionary and developmental theories of social cognition, little is known about the underlying mechanisms. In predictive models of social cognition, a perceptual prediction of an ideal efficient trajectory would be generated from prior knowledge against which the observed action is evaluated, distorting the perception of unexpected inefficient actions. To test this, participants observed an actor reach for an object with a straight or arched trajectory on a touch screen. The actions were made efficient or inefficient by adding or removing an obstructing object. The action disappeared mid-trajectory and participants touched the last seen screen position of the hand. Judgements of inefficient actions were biased towards the efficient prediction (straight trajectories upward to avoid the obstruction, arched trajectories downward towards the target). These corrections increased when the obstruction's presence/absence was explicitly acknowledged, and when the efficient trajectory was explicitly predicted. Additional supplementary experiments demonstrated that these biases occur during ongoing visual perception and/or immediately after motion offset. The teleological stance is at least partly perceptual, providing an ideal reference trajectory against which actual behaviour is evaluated.

## 1. Introduction

Human and non-human primates take the ‘intentional stance’ when watching conspecifics [1], interpreting their behaviour as purposeful and goal-directed [2–6]. Crucial to this is the understanding that others' actions are optimized to achieve their goals in the most efficient and rational way, minimizing time and energy expenditure, given the environmental constraints. Both human infants and macaque monkeys, for example, show surprise when intentional agents do not attempt to avoid an obstacle, or take an unnecessary long way to reach their goal [5,7]. This simple efficient action heuristic can provide a foundation for the development of sophisticated capacities for mentalizing and theory of mind in adult humans (e.g. [5,8]). For example, seeing a seemingly inefficient action (e.g. a reach straight for an object despite an obstacle in the way) can prompt the insight that others may act according to beliefs that differ from one's own (i.e. they may not have seen the obstacle). Indeed, seeing such actions captures attention [9] and alters activity in brain areas implicated in action perception and mentalizing (e.g. [10,11]).

Yet, despite the crucial role of teleological/intentional reasoning in human and animal social cognition, little is known about the underlying processes. The currently dominant view sees social perception as a bottom-up ‘resonance’ of one's own motor apparatus with others' actions, which allows the associated goals and internal states (sensations and emotions) to be derived (e.g. [12,13]). Action efficiency would, in such a model, be conceptualized as a *post hoc* motoric signal of effort or energy expenditure, which can be compared with a reference value for this type of action (e.g. [14]). However, such models are challenged by findings that children make efficiency judgements for movements of biomechanically impossible actions for which motor resonance is unlikely [15], that they can process efficiency before acquiring competence in the seen actions (e.g. [16,17]) or that, in adults, eye movements indicate expectations of efficient action before action onset, when such kinematic information is not yet available [18].

An alternative is that teleological reasoning might not emerge from a ‘late’ motoric signal, but from earlier perceptual signals (e.g. [6]). Recent predictive coding frameworks argue that perception in general—and social perception in particular—is informed by prior expectations, derived from one's knowledge about the world and other people, and that these expectations guide processing of the perceptual input [19–24]. Predictive influences have been demonstrated in a diverse range of perceptual abilities, including the perception of ‘true’ colour from surrounding illumination [25], anticipated effects of physical dynamics on motion perception [26] and three-dimensional (3D) concave/convexity from the presumed location of light sources [27]. In a similar way, the environment provides all the necessary information to generate an ideal reference trajectory that a fully rational, intentional actor would take to achieve their goal (i.e. location of goal objects and possible obstructions), and which would provide a comparison to immediately flag observed actions as being efficient or not, confirming prior attributions of goals and intentionality.

Here, we provide a first test of (i) whether human observers make such predictions of how rational actors who are aware of all environmental constraints efficiently traverse the given action space, (ii) whether these predictions are realized in a perceptual format that can serve as a reference image for the observed action, and (iii) whether this format biases the perceptual representation of the observed action. We rely on the well-established phenomenon that when a moving stimulus suddenly disappears, participants' estimations of its last seen position show robust distortions towards the expected path (i.e. representational momentum [28]), in line with the notion that the considerable uncertainty during motion perception is sharpened by top-down information (e.g. [29,30]), or that predicted paths are perceptually ‘filled in’ after the sudden offset [31]. Importantly, these distortions rely on changes to lower-level visual representations (e.g. [32,33]), occur even when participants are warned against them [34,35] and can integrate higher-level information such as the physical forces acting on the objects (e.g. momentum, friction and gravity; for a review, see [28]) or prior action expectations [36–38].

Here, we use this paradigm to reveal the expectations of efficient action that guide the perception of others' actions. In three studies, participants watched an actor reach towards an object. The action disappeared mid-trajectory, and participants indicated the perceived disappearance point on a touch screen. In two conditions, the actions were efficient, showing a reach either straight towards the object or arched over an obstacle placed in between. In two other conditions, the actions were made inefficient by either adding an obstacle to the path of the straight reaches (such that the actor would knock into the obstacle) or removing the obstacle for the arched reaches (such that the actor reached over empty space). If others’ behaviour is perceived relative to what would be expected under the implicit assumption of efficient action, then the perceived kinematics should be displaced along the trajectory that an intentional, rational actor might take. Unexpected inefficient actions should be ‘corrected’ towards the predicted efficient action trajectory: straight reaches would be perceived upward if approaching an obstacle where an avoidance movement would be predicted, while an arched reach would be displaced downwards if made over empty space as this energy expenditure is unnecessary. Moreover, such distortions should be observed spontaneously when participants passively observe these actions, but should increase the more that attention is drawn to the environmental constraints and the behaviour of a rational actor. As an additional between-subjects task manipulation, we therefore varied whether the actions were viewed under no additional instructions (no task), or whether participants were asked to report ‘yes’ or ‘no’ in response to the presence of an obstacle prior to action onset (report obstacle) or to predict whether a rational actor would ideally have to reach ‘straight’ or ‘over’ an obstacle before the action started (predict trajectory). Further experiments showed that these deviations are not observed when static non-action stimuli have to be judged in the same scenes (electronic supplementary material, Experiment 1), that they generalize to probe judgements tasks that do not require working memory or touch screen responses (electronic supplementary material, Experiment 2) and that they are substantially reduced through dynamic visual noise masks that disrupt recurrent interactions between early visual areas and top-down information (electronic supplementary material, Experiment 3).

## 2. Material and methods

### (a) Participants

Eighty-five participants took part (mean age = 24 years, s.d. = 7.7, 62 females; no task: *n* = 30, report obstacle: *n* = 27, predict trajectory: *n* = 28). Seven additional participants were excluded due to performance (see Results). All participants were right-handed, had normal/corrected vision, were recruited from Plymouth University and wider community, and received course credit or payment. The study received ethical approval from the University of Plymouth's ethics board, in accordance with those of the ESRC and the Declaration of Helsinki. *A priori* power analyses of previous experiments investigating similar effects with the same method ([36], Experiment 3) revealed that a sample size of 14 is required to achieve power of 0.95.

### (b) Apparatus

Stimuli were filmed with a Sony HD video camera at 50 f.p.s. with a widescreen aspect ratio (16 : 9) and a resolution of 1920 × 1080 (2.1 megapixels) and edited with Adobe Photoshop. The experiment was delivered using Presentation (NeuroBS) via an NEC Multisync P221w LCD touch screen monitor (1680 × 1050). Verbal responses for the report obstacle and predict trajectory conditions were recorded using Presentation's sound threshold logic via a Logitech PC120 combined microphone and headphone set.

### (c) Stimuli

**Figure 1.**
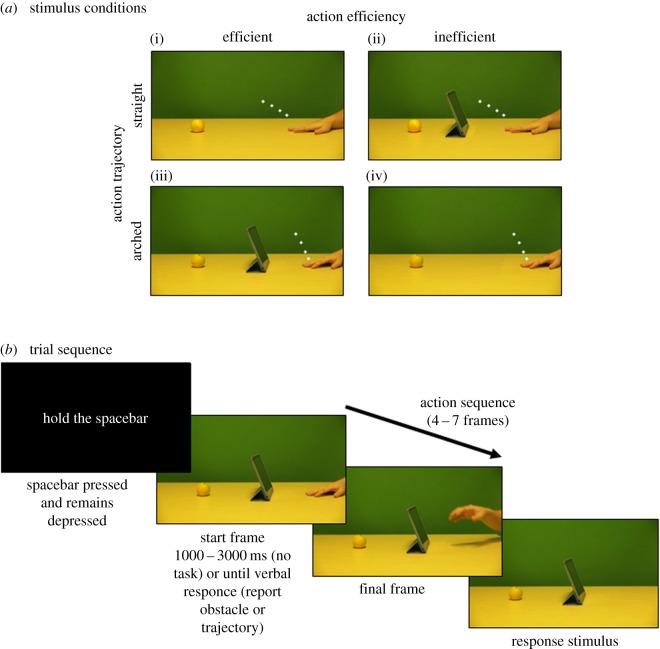
Stimulus conditions and trial sequence. The stimulus conditions are depicted in (*a*). The action trajectory was either straight (i,ii) or arched over (iii,iv). The presence or absence of an obstructing object made the action trajectory either efficient (i,iii) or inefficient (ii,iv). In all examples, the hand is in the initial start position, and the white markers depict the final four frames of the trajectory of the index finger tip. The action sequence disappeared at one of these four points. An example trial sequence is depicted in (*b*), depicting an efficient arched trajectory over an obstruction. (Online version in colour.)

Example stimuli can be seen in figure 1*a*. Videos were filmed of an arm starting in a rest position at the right of the screen and reaching to grasp a target object on the left (either an apple, bottle, crisps, glue stick or stapler). In the original set of videos, the actor's reach was either (i) unobstructed and the trajectory of the arm was straight towards the target object (straight/efficient), (ii) obstructed by one of four objects (iPad, lamp, pencil holder or photo-frame) and the trajectory of the arm was arched over the obstruction (arched/efficient). From each video, 19 frames were extracted for the experimental stimuli, beginning with the onset of movement (frame 1) to mid-way through the action (frame 19). Inefficient action sequences were created by digitally removing the obstructing objects in the arched/efficient videos (arched/inefficient). For each of the straight/efficient actions, a new set of videos were created by adding each of the obstructing objects to show the actor was reaching straight for the target, despite the obstruction (straight/inefficient). This created a set of inefficient actions that were identical to the efficient actions in terms of movement kinematics, and differed only by the presence/absence of the obstructing object. Finally, for each action, a single frame was created in which the hand was digitally removed. This served as a response stimulus at the end of each trial where participants estimated the disappearance point of the action.

### (d) Procedure

Participants completed two blocks of 80 trials in which each combination of action trajectory (straight and arched) and efficiency (efficient and inefficient) was represented by 20 trials. Participants were instructed that, on each trial, they would see an actor reach from the right of the screen for a target object on the far left, but that sometimes there would be a second object in between.

An example trial sequence can be seen in figure 1*b*. At the start of each trial, participants were instructed to ‘hold the spacebar’ and to keep it depressed to prevent them from tracking the observed action with their finger to improve performance. They then saw the first frame of the action sequence as a static image. In the no task condition, the action sequence began after a randomly chosen delay of between 1000 and 3000 ms. In the report obstacle condition and predict trajectory conditions, it began 1000 ms after the participant's verbal response had been detected. In the report obstacle condition, participants said ‘no’ if there was no obstruction and ‘yes’ if there was an obstruction. In the predict trajectory condition, participants said ‘straight’ if there was no obstruction and ‘over’ if there was an obstruction. The action depicted the frame order progressing at three frame intervals for a total sequence of between four and seven frames for 80 ms each (e.g. frames 1–4–7–10–13–16–19). Starting frames and sequence length were randomly chosen on each trial to prevent memorization of the final position from the starting frame. The final frame was immediately replaced by the response stimulus, creating the impression that the hand simply disappeared from the scene. Participants released the spacebar and, with their right hand, touched the screen where they thought the final seen position of the tip of the index finger was. As soon as a response was registered, the next trial began. Debriefing during pilot testing in the report obstacle condition has established that this experimental set-up is relatively opaque to participants and therefore unlikely to capture demand effects (e.g. [39,40]). Participants believed the aim to be investigating speed of motor responses and/or sequence learning, but none of them mentioned action efficiency/rationality or the hypothesized effect of perceptual mis-localizations.

## 3. Results

Participants were excluded if the distance between the real and selected positions exceeded 3 s.d. of the sample mean (mean = 49.2 pixels, s.d. = 12.4, no exclusions), or if the correlation between the real and selected positions was more than 3 s.d. below the median *r* value (*X*-axis: median = 0.914, s.d. = 0.055; *Y*-axis: median = 0.901, s.d. = 0.077, four participants excluded). For each participant, individual trials were excluded if the response procedure was incorrect (spacebar released before the action offset, 4.1%), or if response initiation or execution times were less than 200 ms or more than 3 s.d. above the sample mean (5.1%, initiation: mean = 443.5 ms, s.d. = 84.4; execution: mean = 817.2 ms, s.d. = 230.4). Three additional participants were excluded for having an excessive number of trial exclusions (greater than 50%).

The real final screen coordinate of the tip of the index finger was subtracted from participants' selected screen coordinate on each trial. Analysis was conducted on this residual localization error, which provided a directional measure of how far, in pixels (px), participants' responses were displaced along the *X-* and *Y-*axes. An accurate response would produce a value of 0 on both axes. On the *X*-axis, positive values denote a rightward displacement (against the direction of motion) and negative values a leftward displacement. On the *Y*-axis, positive and negative values denote upward and downward displacements, respectively.

**Figure 2.**
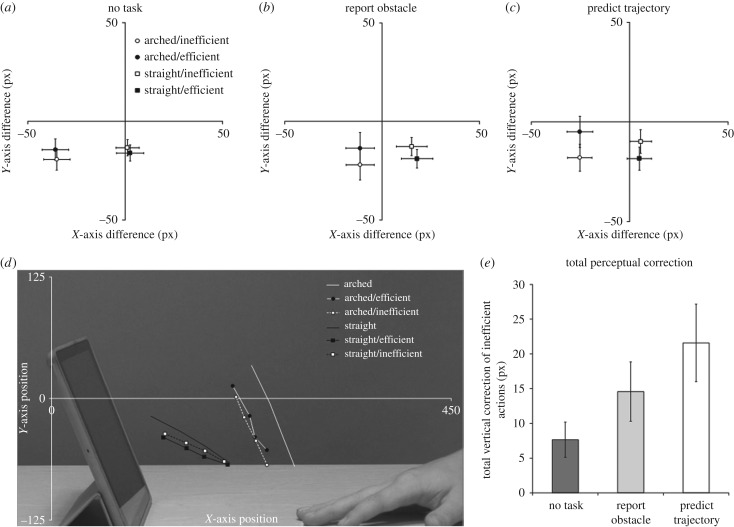
The trajectory × efficiency interactions for each task condition are depicted in (*a*) no task, (*b*) report obstacle and (*c*) predict trajectory. The difference between the real final position and the selected final position is plotted for the *X*-axis and *Y*-axis. The centre of each plot represents the real final position on any given trial (0 px difference on each axis). (*d*) A descriptive representation of the data in real screen coordinates (collapsed across task conditions). The solid lines represent the mean real final position of the arched (white) and straight (black) trajectories for the four possible disappearance points. The selected screen coordinates for each trajectory are plotted for the efficient (filled line) and inefficient (dashed line) conditions. The data are placed over a spatially aligned backdrop of a representative stimulus image of the action start point with an obstructing object to provide a reference of how the data relate to the stimuli. (*e*) A comparison of the size of the *Y*-axis interaction in pixels, equivalent to the total amount by which inefficient actions were corrected towards a more efficient trajectory. Error bars depict 95% confidence intervals.

Overall, there was a significant leftward bias (*X*-axis: mean = −8.4 px, s.d. = 19.2, *t*_84_ = −4.01, *p* < 0.001, *d* = 0.61, 95% CI (−4.3, −12.5)), and a significant downward bias (*Y*-axis: mean = −15.1 px, s.d. = 15.0, *t*_84_ = −9.27, *p* < 0.001, *d* = 1.35, 95% CI (−11.9, −18.3)). The differences along the *X-* and *Y*-axes and the result of the one-sample *t*-test, for each experimental condition across all tasks and for each task individually, can be seen in electronic supplementary material, table S1 (figure 2*a–d*). These difference values were entered into a 2 × 2 × 3 mixed-measures ANOVA for the *X-* and *Y*-axes separately, with trajectory (arched and straight) and efficiency (efficient and inefficient) as within-subjects factors, and task (no task, report obstacle and predict trajectory) as a between-subjects factor.

### (a) *Y*-axis

The main prediction is that perceptual judgements of inefficient actions would be displaced towards the expected trajectory, that is, downwards for inefficient arched trajectories and upwards for inefficient straight trajectories. Indeed, the analysis revealed a main effect of efficiency (*F*_1,82_ = 12.04, *p* = 0.001, 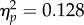) that was qualified by an interaction of efficiency and trajectory (*F*_1,82_ = 136.2, *p* < 0.001, 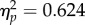). As predicted, inefficient arched trajectories (−19.7 px) were displaced below efficient arched trajectories (−11.0 px, *t*_84_ = −9.33, *p* < 0.001, *d* = 0.47), and inefficient straight actions (−12.0 px) were displaced above efficient straight actions (−17.7 px, *t*_84_ = 8.51, *p* < 0.001, *d* = 0.44), despite the actual hand disappearance points being identical within each trajectory. Importantly, there was a three-way interaction between trajectory, efficiency and task (*F*_2,82_ = 10.6, *p* < 0.001, 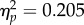). The interaction effect was re-quantified as a single value for each participant ((arched/efficient − arched/inefficient) − (straight/efficient − straight/inefficient)) to reveal the total amount in pixels by which inefficient actions were corrected towards a more efficient trajectory for each task (figure 2*e*). Between-subjects *t*-tests on this interaction value—mathematically equivalent to the pairwise three-way interactions of trajectory, efficiency and task—show that the interaction was marginally larger in the predict trajectory condition than the report obstacle condition (*t*_53_ = −1.95, *p* = 0.057, *d* = 0.53), which in turn was significantly larger than in the no task condition (*t*_55_ = −2.81, *p* = 0.007, *d* = 0.73). Demonstrating the robustness of the interaction, exploratory analysis showed that the interaction of trajectory and efficiency was evident in all conditions with a corrected *α*-level of *p* = 0.017 (no task: *F*_1,29_ = 35.3, *p* < 0.001, 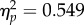; report obstacle: *F*_1,26_ = 44.5, *p* < 0.001, 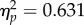; predict trajectory: *F*_1,27_ = 57.7, *p* < 0.001, 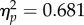; figure 2*a–c*). There were no further main effects or interactions.

### (b) *X*-axis

We did not have specific predictions about how action rationality would affect perceptual displacements on the *X*-axis, and the analysis indeed did not reveal either a main effect of efficiency (*F*_1,82_ = 0.837, *p* = 0.363, 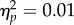) or an interaction of efficiency and trajectory (*F*_1,82_ = 1.39, *p* = 0.242, 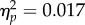). We report the remaining effects of no interest below, but due to *α*-inflation of unpredicted effects in an ANOVA [41], they should be considered exploratory and interpreted with caution. A main effect of task (*F*_2,82_ = 8.81, *p* < 0.001, 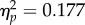) revealed a general leftward displacement in the no task (−16.8 px, *t*_29_ = −5.14, *p* < 0.001, *d* = 1.35) and predict trajectory conditions (−10.0 px, *t*_27_ = −3.43, *p* = 0.002. *d* = 0.86), but not in the report obstacle condition (9 px, *t*_26_ = 0.720, *p* = 0.478, *d* = 0.19). A main effect of trajectory (*F*_1,82_ = 1231.4, *p* < 0.001, 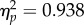) showed a leftward displacement for arched trajectories (−24.4 px, *t*_84_ = −11.0, *p* < 0.001, *d* = 1.7) and a rightward displacement for straight trajectories (7.7 px, *t*_84_ = 3.74, *p* < 0.001, *d* = 0.53), most likely reflecting the further right displaced centre of gravity of the straight arm configurations ([42]; see also [36]). Finally, an interaction between trajectory and task (*F*_1,82_ = 9.88, *p* < 0.001, 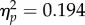) revealed that the trajectory effect was larger in the no task than in the report obstacle (*t*_55_ = 4.15, *p* < 0.001, *d* = 1.1) and predict trajectory conditions (*t*_56_ = 3.25, *p* = 0.002, *d* = 0.86), which did not differ from each other (*t*_53_ = 1.18, *p* = 0.243, *d* = 0.32). There were no further interactions (all values of *p* > 0.351).

### (c) Non-biological stationary stimuli (see electronic supplementary material, Experiment 1)

An alternative explanation for the *Y*-axis interaction between trajectory and efficiency on perceptual judgements is that the presence of an obstacle reduced the amount of general downward displacement. In electronic supplementary material, Experiment 1 (reported in full in the electronic supplementary material), the hands were therefore replaced by a static geometric shape in the same position as the disappearance points of the task relevant index finger, which crucially could not be interpreted in terms of intentionality nor efficiency. However, here, the presence/absence of an obstacle had no effect on these perceptual non-action judgements.

### (d) Replication with probe judgements (see electronic supplementary material, Experiment 2)

Electronic supplementary material, Experiment 2 (reported in full in the electronic supplementary material) confirmed that the perceptual shifts towards efficient actions could also be observed in a psychophysical task without motor or working memory components. Here, participants simply reported—with a press of a button—whether the index finger's seen disappearance was identical to a probe stimulus presented directly after action offset (250 ms gap to prevent masking), which could be displaced either subtly upwards or downwards from the real disappearance point. Mirroring the results of the main experiments, participants were more likely to misidentify probes displaced towards the predicted ‘ideal’ trajectory with the actually perceived disappearance point (*F*_1,36_ = 11.39, *p* = 0.002, 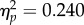). They more readily accepted downwards probes as the same as the last seen position of inefficient arched reaches, and upwards probes for inefficient straight reaches. This replication rules out that the effects emerge from perceptual changes to the action's representation in later working memory or motor control stages, and instead reveals a contribution to immediate perceptual processing, either during ongoing motion perception (e.g. [43,44]) or to perceptual ‘filling in’ in the brief interval directly after its sudden offset (e.g. [31]).

### (e) Masking with dynamic visual noise (see electronic supplementary material, Experiment 3)

Electronic supplementary material, Experiment 3 (reported in full in the electronic supplementary material) tested whether the perceptual shifts towards the efficient trajectories can be disrupted with a short (560 ms) dynamic visual noise mask directly after action offset that was presented in 50% of trials, at the same frequency as the prior motion stimuli (80 ms per frame), while maintaining equivalent task demands as the main experiment. Such masks reliably disrupt lower-level perceptual processes [45,46], eliciting similar effects to transcranial magnetic stimulation of occipital cortices (e.g. [47]). They specifically interrupt re-entrant interactions between V1 and higher visual areas that are crucial for conscious access to a stimulus during actual perception (e.g. backwards masking [48,49]) or visual imagery (e.g. [50,51]). Indeed, while the non-mask trials fully replicated the perceptual shifts towards efficient actions, they were reduced to about one-third of their size in the masked trials (*F*_1,26_ = 8.89, *p* = 0.006, 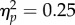). These masking effects therefore further confirm that the perceptual biases in the main experiments reflect changes to early visual stimulus representation, specifically tying them to the re-entrant feedback required for stabilizing percepts in perception or visual imagery (e.g. [48,51]).

## 4. Discussion

The present study showed for the first time that the teleological interpretation humans have of others' behaviour is perceptually instantiated and provides a visual reference signal for an expected ‘ideal’ trajectory during action observation. Participants watched a hand reach for objects with either efficient or inefficient kinematics and reported its last position after it had suddenly disappeared. Across several samples, perceptual reports were consistently biased towards the ideal reference kinematics. Straight reaches were reported higher if there was an obstacle in the way, as if lifted to avoid it. Conversely, reaches with a high arched trajectory were reported lower if the path was clear. These biases were evident automatically, but became more pronounced when observers explicitly processed the potential obstacles that could constrain the action and particularly when they predicted the most efficient action kinematics through the scene.

Further experiments showed that the biases in perceptual judgements were action-specific and not elicited when locations of briefly presented non-biological stationary objects were judged in the same scenes (electronic supplementary material, Experiment 1). They could also be observed in a probe judgement task without working memory or motor components already at 250 ms after action offset (electronic supplementary material, Experiment 2), did not increase with longer response times (electronic supplementary material, response time (RT) analysis) and were effectively disrupted by dynamic visual noise masks (electronic supplementary material, Experiment 3), which interfere with the recurrent (top-down) feedback to early visual cortex [48], preventing its use in awareness (i.e. backwards masking, [46,47]) or visual imagery (e.g. [50,51]). The observed biases in perceptual judgements are therefore unlikely to stem from unspecific perceptual changes in memory or motor control (e.g. [40]; see [52] for an example for perceptual changes in action memory). Instead, they support a role in ongoing motion perception (e.g. [43]) or occurring directly after its sudden offset, when the predicted future trajectory is visually ‘filled in’ (e.g. [31]).

The results are also unlikely to reflect the cognitive/social demands of the experimental set-up (e.g. [39,40]). The rapid nature of action sequences and resulting touch responses precluded considered decision-making that takes into account the required pattern across experimental conditions. Moreover, the effect was replicated in a more complex and cognitively opaque probe judgement task (electronic supplementary material, Experiment 2), which is largely unaffected by conscious strategic manipulation [34,35]. Finally, even though cues to the research question were equivalent, the perceptual biases were successfully reduced by brief dynamic visual noise masks directly after stimulus offset (electronic supplementary material, Experiment 3), which are known to disrupt perceptual processes specifically [48,49].

Together, these results reveal that, during social perception, the principle of efficient action provides a similar perceptual reference signal to the assumption that light comes from above [27] or that gravity pulls objects downwards [53], constantly pushing the perceptual representation of inefficient actions towards a more rational path. The resulting biases in perceptual judgements cannot be accounted for by an abstract awareness of the action's goal, such as when eye movements jump towards an action's target [54] or perceptual judgements are biased towards them [36–38]. Instead, they reveal concrete expectations of the specific trajectory that the action will take through the scene. Moreover, making this awareness explicit prior to action observation increased the perceptual bias. Action efficiency is therefore not only evaluated after an action has been completed [5,7,9,55], but constantly updated, at every step in the trajectory, by predictions that can be derived from contextual cues prior to the motor behaviour.

Our results support predictive coding frameworks of social perception [19–24], which argue that social perception, like perception in general, is hypothesis-driven and guided by top-down expectations. In such models, observers constantly test their inferences about others' goals and beliefs by predicting how they would behave, and matching this prediction to—and integrating it with—the actual perceptual input. In such a view, predictions of efficient action can contribute to the perceptual sharpening of the visual uncertainty during action perception (i.e. motion blurring, [29]), or after its offset, constantly biasing perception towards the expected avoidance or straightening movement, with the amount of bias constrained by the visual uncertainty. In addition, they allow humans to rapidly confirm the intentionality of others' behaviour, only requiring a match of the observed actions to the ideal kinematics that would follow from these goals. Prediction errors, by contrast, would signal inefficient actions, triggering more sophisticated mentalizing processes to re-evaluate the actor's goal or how their beliefs may differ from one's own [5,10,11]. In this way, the relatively simple perceptual process of prediction and prediction error would not only support perception, but also provide a foundation for higher-level judgements about others' beliefs or intentions, even in cases in which motor experience is unlikely [15–17].

An important question is at which level the present predictive biases on social perception arise. While attribution of goals is often seen as a higher-level process (e.g. [19,23,24]), the detection of intentionality has been argued to be a feature of perceptual processing itself, based on specific stimulus features that signal intentionality and which allow humans to ‘see’ the agency behind others' movements (for a review, see [6]). A perceptual expectation of efficient action could emerge directly from such low-level unconscious perceptual inferences. Indeed, a series of follow-up studies ([56]; pre-print at: https://doi.org/10.17605/OSF.IO/QWJTF) has linked the perceptual displacements to stimulus features that imply intentional action, being almost completely eliminated for moving stimuli that are not intentional (e.g. a ball) and do not follow the characteristic motion profile of intentional action towards objects (e.g. [57]). Such a perceptual origin is also consistent with the finding that, in humans, sensitivity to kinematic efficiency emerges early in development, that it is present in other primates [7] and that it is spared in individuals with autism spectrum conditions, for whom only more advanced mental state reasoning proves problematic [9,58]. The key leap to the sophisticated socio-cognitive abilities of humans may therefore lie in the abstraction of these lower-level content-based representations of goals, environment and action, to higher-order representations of desires, beliefs and intentions, respectively [5]. For example, it is clear that human infants from the age of 4 onwards are able to predict what others will do not based on the actual environmental constraints (toy is in box A), but in terms of what the actor believes the state of the environment to be (they believe it is in box B), suggesting that the bottleneck emerges at this later state that requires sophisticated coordination of representation, such as others’ beliefs that differ from one's own beliefs or objective reality [59–61].

## 5. Conclusion

The principle of efficient action allows observers to perceive others' actions relative to ideal reference actions, thereby confirming prior goal attributions or revising them in the case of a conflict. Such perceptual mechanisms for efficiency perception support rapid attribution of intentionality and facilitate the perception of others’ behaviour and our interactions with them. The burden of social cognition is placed on mechanisms that account for unexpected behaviour through a re-evaluation of their beliefs, desire and intentions, so that our model of the social world can be refined, and predictions of other's behaviour can be made more accurately.

## Supplementary Material

Supplementary Analysis, Experiments, and Tables

## Data accessibility

A summary of the data for each experiment is provided in an Excel file as the electronic supplementary material and available in the Dryad Digital Repository: https://doi.org/10.5061/dryad.m9j93jg [62].
