# Supplementary material for: Perceptual teleology: expectations of action efficiency bias social perception
Source: Proc Biol Sci. 2018 Aug 8;285(1884):20180638. doi: 10.1098/rspb.2018.0638 (PMC6111183; doi:10.1098/rspb.2018.0638)
Supplement: Supplementary Analysis, Experiments, and Tables [file rspb20180638supp1.pdf]

Perceptual Teleology: Expectations of Action Efficiency Bias Social Perception.

Proceedings of the Royal Society B: Biological Sciences

http://doi.org/10.1098/rspb.2018.0638

Matthew Hudson[1], Katrina L. McDonough[1], Rhys Edwards[1], Patric Bach[1]

[1]University of Plymouth, UK

Electronic Supplementary Material

Data available from: Hudson M, McDonough K, Edwards R, Bach P. (2018). Data from: Perceptual teleology: expectations of action efficiency bias social perception. Proceedings of the Royal Society B https://doi.org/10.5061/dryad.m9j93jg

Supplementary Analysis: The effect of action efficiency on response time, and its relation to the spatial displacement.

We examined the effect of action efficiency on the initiation (time between test stimulus onset and spacebar release) and execution (time between spacebar release and touch screen contact) of responses, and how this temporal variability may relate to the extent of the spatial displacements.

Response initiation times did not differ between efficient and inefficient actions (F(1,82) = .084, p = .772, η_p^2 = .001), but were quicker for straight actions (M = 438.4 ms, SD = 85.5) than for arched actions (M = 451.1 ms, SD = 85.6) (F(1,82) = 14.3, p < .001, η_p^2 = .148). A Trajectory X Efficiency interaction (F(1,82) = 5.41, p = .022, η_p^2 = .062) revealed that, for arched actions, response initiation was quicker for efficient (M = 447.6 ms, SD = 83.9) than inefficient trajectories (M = 454.5 ms, SD = 92.0), but for straight actions, quicker for inefficient (M = 435.7 ms, SD = 85.8) than efficient trajectories (M = 441.1 ms, SD = 88.2). A three-way Trajectory X Efficiency X Task interaction (F(1,82) = 5.33, p = .007, η_p^2 = .115) showed that the Trajectory X Efficiency interaction was smaller in the Report Obstacle condition than the Predict Trajectory or No Task conditions.

Response execution times were similarly equivalent for efficient and inefficient actions (F(2,82) = .172, p = .679, η_p^2 = .002), and also showed a Trajectory X Efficiency interaction (F(1,82) = 10.5, p = .002, η_p^2 = .114), but in the opposite direction. Execution times were quicker for inefficient (M = 817.0 ms, SD = 232.6) than efficient Arched actions (M = 826.8 ms, SD = 234.3), but quicker for efficient (M = 812.6 ms, SD = 228.1) than inefficient straight actions (M = 825.3 ms, SD = 231.5).

Initiation and execution times were binned in 20% increments according to the participant's response time distribution. The interaction size was calculated for each response time bin for each participant and entered into a one-way ANOVA with time bin as a within

subjects factor. The Trajectory X Efficiency interaction did not differ with response initiation time bin (F(4,336) = .855, p = .491, η_p^2 = .010). There was a marginal effect of Bin on response execution times (F(4,336) = 2.26, p = .063, η_p^2 =.026), with a trend for the Trajectory X Efficiency interaction to decrease with increasing execution times.

Supplementary Experiment 1: Non-biological stationary stimuli

An alternative interpretation of the Trajectory X Efficiency interaction observed so reliably in the main experiments is that of a general effect of obstacle presence. That is, the overall downward displacement may have simply been reduced when an obstacle was present (straight/inefficient, arched/efficient) than when it was absent (straight/efficient, arched/inefficient). This low-level interpretation was tested explicitly in this control experiment. The biological hand stimuli of the main experiments were replaced by a circle of the same colour and size as the tip of the index finger. The placement of the circle was matched to the final position of the index finger in the videos of the main experiments, for both Straight or Arched trajectories, with or without obstacle being present. However, the circle remained stationary so as not to create the impression of animacy or agency that even simple geometric shapes can generate in observers (Heider and Simmel, 1944; Ramsey & Hamilton, 2010). Therefore, the location that participants were required to respond, and its spatial relationship with the obstacle and target object, were equivalent to the main experiments, yet crucially the stimulus position could not be interpreted in terms of intentionality or its (in)efficiency with respect to the absence/presence of the obstruction.

To further maximise the potential of replicating the effect, prior to stimulus onset participants said “Yes” or “No” depending on the presence/absence of the obstacle, as in the Report Object task of the main experiments. This ensured that participants explicitly processed the obstructing object, which enhances the perceptual bias. If the results of the main experiments are indeed due to a prediction of action efficiency biasing the perception of unexpectedly inefficient actions, then no such predictions should be generated for the location of a stationary geometric shape, and no perceptual bias should be evident. However, as the presence or absence of an obstructing object remains, a perceptual bias should still be evident if the results can be explained by a low-level facilitative effect.

Method

Participants

Fifteen participants took part in the experiment (mean age = 32.1 years, SD = 15.7, 12 females, 12 right handed). All had normal/corrected vision, were recruited from Plymouth University and wider community, and received course credit or payment. As participants completed twice the number of trials as those in the main experiments (see Procedure), a reduced sample size could be tested whilst maintaining an equivalent level of statistical power.

Apparatus & Stimuli

All apparatus are the same as in the main experiments. The target stimulus was a circle the same size (30 X 30 px) and colour as the tip of the index finger of the action stimuli in the main experiments.

Procedure

The design of the experiment matched that of the main experiments. Each of the 80 different movie sequences were represented, with the placement of the circle corresponding to the four final positions in each respective movie, producing 320 trials in total. The duration of the circle was aligned to the duration of the action stimulus. For example, a position that matched an offset after 4 frames was on screen for 320 ms (4 x 80 ms), whereas a position that matched an offset after 7 frames was onscreen for 560 ms (7 x 80 ms). As in the main experiments, each trial began with the instruction to hold the spacebar, after which an image depicting the target object on the far left and, when relevant, the obstructing object (the response stimulus from the main experiments) was shown, to which participants responded either “Yes” or “No”. After a delay of between 1000 – 3000 ms, the circle appeared and

disappeared. Participants then released the spacebar and touched the screen where they thought the circle had appeared, after which the next trial began.

Results

Participant performance and exclusions

No participants were excluded on the basis of the distance between the real and selected screen coordinate (mean = 22.5 px, *SD* = 4.5), but one was excluded based on the correlation between the real and selected positions on the X (median r = .991, *SD* = .028) or Y axis (median r = .987, *SD* = .029).

Due to the stationary nature of the stimulus, anticipatory responses (releasing the spacebar before stimulus offset) were excessively high (28.8%). Furthermore, the number of trials in which a response was initiated less than 200 ms after stimulus offset (31.1%) was considerably higher than in the main experiments (3.5%). To maintain equivalent trial numbers, the lower limit of 200 ms for the inclusion of trials based on response initiation times was removed, such that only responses initiated more than 3SD slower than the group mean were excluded (the results were unaffected by this altered exclusion criteria). Response executions times were comparable to those of the main experiments (*M* = 784.8 ms, *SD* = 190.8) and were excluded based on the same criteria as the main experiments. Overall, 0.6% of trials were excluded.

Data analysis

As the positions of the stationary circles matched those of the different action trajectory conditions of the main experiments, it was possible to analyse the screen coordinates of the stimulus in terms of Action Trajectory (straight, arched) and Action

Efficiency (efficient, inefficient), an interaction of which is equivalent to a main effect of obstacle presence/absence, to facilitate comparison with the main experiments.

Y Axis

Overall, there was a significant downward bias (*M* = -10.4px, *SD* = 3.4, *t*(13) = 11.4, $p < .001$, $d = 1.08$). There was a main effect of Action Efficiency ($F(1,13) = 5.08$, $p = .042$, $\eta_p^2 = .281$), with inefficient actions eliciting a larger downward displacement (*M* = -11.0px, *SD* = 3.1, *t*(13) = 13.3, $p < .001$, $d = 1.28$) than efficient actions (*M* = -9.8, *SD* = 3.9, *t*(13) = 9.3, $p < .001$, $d = .91$). There was no main effect of Action Trajectory ($F(1,13) = .643$, $p = .437$, $\eta_p^2 = .047$) and, importantly, no interaction between Action Trajectory and Efficiency ($F(1,13) = 2.48$, $p = .139$, $\eta_p^2 = .160$).

X Axis

There was a significant overall rightward bias (*M* = 13.9px, *SD* = 3.0, *t*(13) = 17.0, $p < .001$, $d = 1.88$). A main effect of Action Trajectory ($F(1,13) = 11.2$, $p = .005$, $\eta_p^2 = .462$) showed that Straight actions elicited a larger rightward displacement (*M* = 14.5px, *SD* = 3.0, *t*(13) = 18.0, $p < .001$, $d = 1.62$) than Arched actions (*M* = 13.3px, *SD* = 3.2, *t*(13) = 15.4, $p < .001$, $d = 2.04$). There was no main effect of Action Efficiency ($F(1,13) = .023$, $p = .883$, $\eta_p^2 = .002$), nor an interaction between action Trajectory and Efficiency ($F(1,13) = .330$, $p = .576$, $\eta_p^2 = .025$).

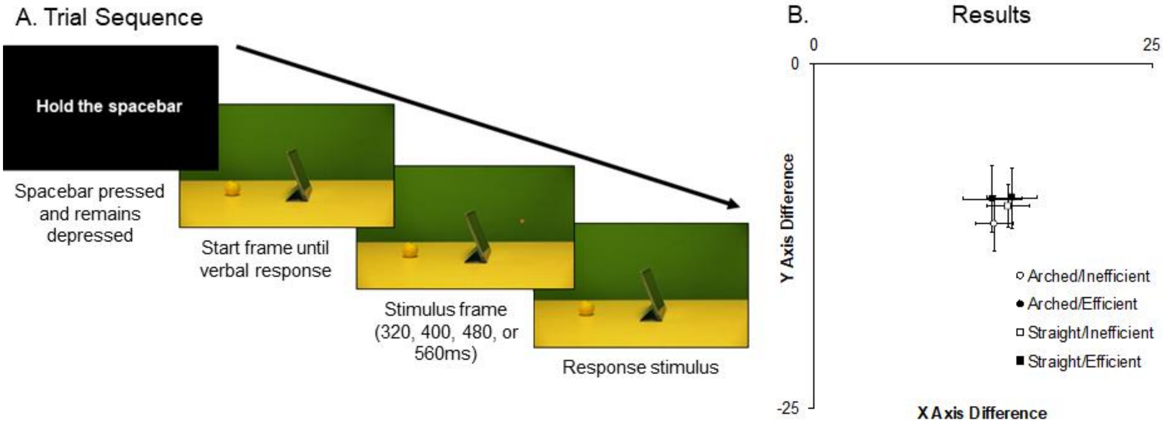

Supplementary Figure 1. Trial sequence and results for Supplementary Experiment 1. An example of the trial sequence is depicted in Panel A, with the action sequence replaced by a stationary circle that matched the tip of the index finger for size, colour and position. The results are depicted in Panel B. The difference between the real location and the selected location is plotted along the X and Y axis. A value of 0 on both axis indicates no difference, and therefore the real position on any given trial. Despite the stimulus being a stationary circle, the locations reflected the 4 stimulus conditions (Trajectory X Efficiency) of the main experiments, which are depicted here to facilitate comparison. Error bars represent 95% confidence intervals.

Discussion

The results demonstrate that when participants were required to localise the screen position of a stationary geometric shape, the presence or absence of a second object did not influence participant responses. The perceived location was no more upwards when the “obstruction” was present than when it was absent. These results are very different to those of the main experiments. This implies that the observed perceptual biases very much rely on the participant’s interpretation of the action as goal directed, and that the second object is acting as an obstruction that determines whether that action is efficient or not. Neither interpretation is available when the stimulus to be localised is a simple geometric shape.

References

Heider, F., & Simmel, M. (1944). An experimental study of apparent behavior. *The American Journal of Psychology, 57,* 243-259. Doi:10.2307/1416950

Ramsey, R. & Hamilton, A. F. de C. (2010). Triangles have goals too: Understanding action representation in left aIPS. *Neuropsychologia, 48,* 2773-2776. Doi:10.1016/j.neuropsychologia.2010.04.028

Supplementary Experiment 2: Probe judgments

The touch screen judgements of the main experiments provide a direct measure of perceptual shift in each trial, but leave open at which processing step they occur. Do they directly affect the perceptual representations of the observed actions, or do they emerge from later changes to the action's perceptual representations in working memory or in the sensorimotor maps that guide the motor responses to the relevant locations on the screen? Here, we therefore replicate the Report Object experiment with a well-established psychophysical task that is free from such memory or motoric influences, but reliably measures changes to the perceived motion in the predicted path (i.e., representational momentum, Freyd & Finke, 1984; Hudson, Bach & Nicholson, 2017; Hudson, Nicholson, Ellis & Bach, 2016; Hudson, Nicholson, Simpson, Ellis & Bach, 2016, for reviews, see Hubbard, 2005; Kerzel, 2005).

In each trial, participants compared the hand's last seen position to a probe stimulus presented directly after hand offset (250 ms gap), which was displaced vertically either in the predicted direction (e.g. downwards for inefficient arched reaches) or in the opposite unpredicted direction, and horizontally leftwards or rightwards. Participants indicated, with the press of a button, whether the probe stimulus position was identical or different from the hand's last seen position on the screen. Importantly, if predictions of efficient action affect the on-going perceptual representation of the observed actions (for example in non-biological perception, see Muckli, Kohler, Kriegeskorte & Singer, 2005; Yantis & Nakama, 1998) or lead to spontaneous perceptual filling in of the predicted trajectories after the sudden offset (e.g., Ekman, Kok & de Lange, 2017), then participants should be more likely to mistake probe displacements in the expected direction with the hand's last seen position, compared to displacements in the opposite direction. Because the probe stimuli appear directly after action offset and participants' responses do not need access to visuospatial representations, any such

effects will therefore reflect either perceptual changes during on-going action observation or directly after action offset.

Method

Participants

Thirty-nine participants took part in the experiment (mean age = 20.0 years, SD = 1.7, 28 females). All participants were right-handed, had normal/corrected vision, and were recruited from Plymouth University for course credit.

Apparatus & Stimuli

The experiment was presented on a HP EliteDisplay S230tm 23-inch widescreen (1920 X 1080) touch screen monitor. Verbal responses were recorded with Microsoft LifeChat LX-3000 Headsets. All other components of the apparatus were the same as in the main experiments. The stimulus set was identical to the main experiments. The only addition was the probe stimulus, a single red circle the same size (30 X 30 pixels) as the tip of the index finger of the action stimuli in the main experiments.

Procedure

The design of the experiment closely matched that of the main experiments. As before, participants completed two blocks of 80 randomised trials. Each trial began with the first static image of the action sequence, and continued to replicate the trial sequence of the Report Obstacle experiment until the response stimulus. Thus, participants saw the action commence after they reported, verbally into the microphone, whether an obstacle was present in the scene. After the action disappeared, participants did not make a touch response. Instead, the probe stimulus was presented 250 ms after hand offset (preventing masking

effects, Breitmeyer & Öğmen, 2006). The probe stimulus was overlaid on top of the scene (without hand) and was positioned at either the same coordinates as the tip of the index finger, or at one of 12 different positions. These positions were derived from the average displacements induced by inefficient compared to efficient actions recorded in the Report Obstacle experiment (X = 24.3 pixels, Y= 19.2 pixels). Four of the different positions were calculated as the coordinates of the tip of the index finger, plus or minus 50% of these average displacement pixels, 100% of the average displacements, or 150%. Panel B of Supplementary Figure 2 depicts all 13 possible probe positions. Participants were required to press the spacebar if they judged the probe to be in a position different to the tip of the index finger and do nothing if they judged it to be in the same position.

Each participant received two practice blocks containing six trials each. In the first practice block, the final action frame remained on screen instead of the response stimulus, and the probe was overlaid on top of this frame. This made it clear to participants when the probe was in the same or different position as the tip of the index finger. The second practice block was the same as the experimental trials.

Results

Participant performance and exclusions

Participants were excluded if the correlation between their probe judgements and the probe positions was more than 3SD away from the median r value (X axis: median = .858, *SD* = .141; Y axis: median = .898, *SD* = .123, 2 participants excluded). Exclusion of these participants does not affect the results. Individual trials were excluded if response times were faster than 200 ms or slower than 3000 ms (.04% of trials).

Data analysis

Analysis was conducted on the proportion of “different” responses, averaged across the three probe positions in each of the four directions. Difference scores were calculated along the X and Y axis separately to measure the size of the perceptual shift. For the X axis, responses for rightward probes were subtracted from responses for leftward probes. Therefore, positive difference scores denote the proportion of rightward probes judged as “same” and negative difference scores denote the proportion of leftward probes judged as “same”. For the Y axis, responses for upward probes were subtracted from responses for downward probes. Therefore, positive difference scores denote the proportion of upward probes judged as “same” and negative difference scores denote the proportion of downward probes judged as “same”. These difference scores were entered into two separate 2 X 2 ANOVAs with Trajectory (arched, straight) and Efficiency (efficient, inefficient) as within-subjects factors.

Y Axis

Overall, there was a main effect of Trajectory ($F(1,36) = 13.44$, $p = .001$, $\eta_p^2 = .272$), where the likelihood to accept upward compared to downward probes as “same” was greater for arched reaches (-.004) than for straight reaches (-.07, $t(36) = 3.67$, $p = .001$, $d = 0.60$), consistent with a further extrapolation of the prior motion along its path. A main effect of Efficiency ($F(1,36) = 5.66$, $p = .023$, $\eta_p^2 = .136$) indicated that the likelihood to accept upward compared to downward probes as “same” was greater for inefficient reaches (-.01) than for efficient reaches (-.06, $t(36) = 2.38$, $p = .023$, $d = 0.39$). Most importantly, the analysis revealed the predicted interaction between Trajectory and Efficiency ($F(1,36) = 11.39$, $p = .002$, $\eta_p^2 = .240$). Participants were more likely to accept upwards probes for inefficient straight reaches than for efficient straight reaches ($t(36) = 4.57$, $p < .001$, $d = .94$),

and, numerically, to accept downwards compared to upwards probes as “same” for inefficient arched reaches than for efficient arched reaches ($t(36) = 1.12$, $p = .269$, $d = .26$). These results therefore fully replicate the perceptual shifts towards the efficient trajectories in the main experiment with a psychophysical judgment task without working memory or motor component.

X Axis

As in the main experiment, we did not have specific predictions for the X Axis. The reported effects should therefore be considered exploratory and interpreted with caution. Overall, there was a main effect of Trajectory ($F(1,36) = 47.01$, $p < .001$, $\eta_p^2 = .566$). The likelihood to accept rightward compared to leftward probes as “same” was greater for arched reaches (.08) than for straight reaches (-.05, $t(36) = 6.86$, $p < .001$, $d = 1.23$), most likely reflecting a greater expectation of forward momentum (leftward direction) for straight reaches compared to arched reaches (Representational Momentum; Hubbard, 2005). Interestingly, the analysis revealed an interaction between Trajectory and Efficiency ($F(1,36) = 7.49$, $p = .010$, $\eta_p^2 = .172$), showing that the likelihood to accept rightwards compared to leftward probes as “same” was greater for efficient arched reaches than for inefficient arched reaches, and greater for inefficient straight reaches than for efficient straight reaches. While unpredicted, this finding is fully in line with the expected deviation towards the predicted “efficient” trajectory. Because straight reaches exert more forward displacements than arched reaches (see above), this forward displacement also takes place – albeit to a smaller extent – when participants see an arched reach but predict a straight reach, or conversely, is reduced when participants see a straight reach but predict an arched one. As noted, this effect was not predicted and not observed with the touch screen responses. It should therefore be interpreted with caution before being replicated.

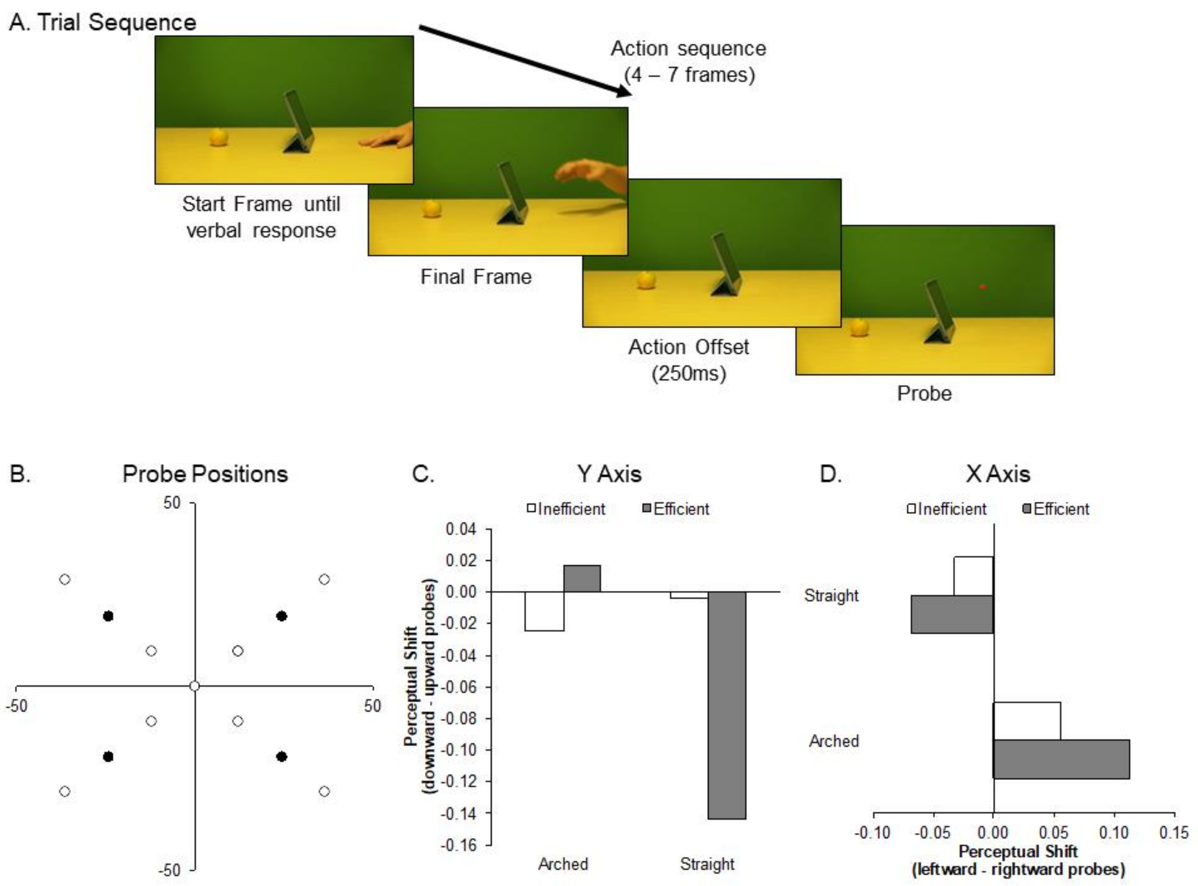

Supplementary Figure 2. Trial sequence, Probe positions and Results for Supplementary Experiment 2. An example of the trial sequence is depicted in Panel A. Panel B depicts all probe positions relative to the final position of the hand, where 0,0 depicts a probe in the same position as the hand. Filled circles depict average displacement pixels as recorded in the Report Obstacle Experiment and empty circles depict plus or minus 50%. The results for the Y axis are depicted in Panel C and the results for the X axis are depicted in Panel D.

Discussion

The results of Supplementary Experiment 2 confirm that perceptual distortions of observed actions towards an ideal reference trajectory can be measured with probe stimuli shortly after action offset (250 ms), with responses that do not rely on perceptual working memory representations or visuospatial motor maps (e.g., Kerzel, 2005). Moreover, because the perceptual biases measured in this paradigm are to a large extent involuntary (Courtney & Hubbard, 2008; Ruppel, Fleming & Hubbard, 2009), they rule out strategic responses aimed to satisfy the experimental demands of the task. The results therefore confirm that the perceptual

changes happen either during on-going motion perception (e.g., Muckli et al., 2005; Yantis & Nakama, 1998), or in the brief interval directly after its sudden offset, when the visual system spontaneously fills in the further expected trajectory (e.g., Ekman et al., 2017). They link the effects either to top-down changes that sharpen the considerable perceptual uncertainty during motion perception (i.e. motion blurring & sharpening, Hammett, 1997), and/or to changes in short term iconic memory that rely on early visual representation and are responsible for their conscious representation, linked to such phenomena as integration of stimulus features, change blindness, and the experience of stable percepts across saccades (e.g., Becker, Pashler & Anstis, 2000; Jonides, Irwin & Yantis, 1982, see Öğmen & Herzog, 2016 for a recent review).

References

Becker, M. W., Pashler, H., & Anstis, S. M. (2000). The role of iconic memory in change-detection tasks. *Perception, 29*, 273-286. Doi:10.1068/p3035

Breitmeyer, B. & Öğmen, H. Visual masking: time slices through conscious and unconscious vision. Oxford University Press, Oxford, 2006. Doi:10.1093/acprof:oso/9780198530671.001.0001

Courtney, J. R., & Hubbard, T. L. (2008). Spatial memory and explicit knowledge: An effect of instruction on representational momentum. *The Quarterly Journal of Experimental Psychology, 61,* 1778-1784. Doi:10.1080/17470210802194217

Ekman, M., Kok, P., & de Lange, F. P. (2017). Time-compressed preplay of anticipated events in human primary visual cortex. *Nature Communications, 8,* 15276. Doi:10.1038/ncomms15276

Freyd, J. J., & Finke, R. A. (1984). Representational momentum. *Journal of Experimental Psychology: Learning, Memory, and Cognition, 10,* 126-132. Doi:10.1037/0278-7393.10.1.126

Hammett, S. T. (1997). Motion blur and motion sharpening in the human visual system. *Vision Research, 37,* 2505-2510. Doi:10.1016/S0042-6989(97)00059-X

Hubbard, T. L. (2005). Representational momentum and related displacements in spatial memory: A review of the findings. *Psychonomic Bulletin & Review, 12,* 822-851. Doi:10.3758/BF03196775

Hudson, M., Bach, P., & Nicholson, T. (2017). You said you would! The predictability of other's behavior from their intentions determines predictive biases in action perception. *Journal of Experimental Psychology: Human Perception and Performance, 44*, 320-335. Doi:10.1037/xhp0000451

Hudson, M., Nicholson, T., Ellis, R., & Bach, P. (2016). I see what you say: Prior knowledge of other's goals automatically biases the perception of their actions. *Cognition, 146,* 245-250. Doi:10.1016/j.cognition.2015.09.021

Hudson, M., Nicholson, T., Simpson, W. A., Ellis, R., & Bach, P. (2016). One step ahead: The perceived kinematics of others' actions are biased toward expected goals. *Journal of Experimental Psychology: General, 145,* 1-7. Doi:10.1037/xge0000126

Jonides, J., Irwin, D. E., & Yantis, S. (1982). Integrating visual information from successive fixations. *Science, 215,* 192-194. Doi:10.1126/science.7053571

Kerzel, D. (2005). Representational momentum beyond internalized physics: Embodied mechanisms of anticipation cause errors in visual short-term memory. *Current Directions in Psychological Science, 14,* 180-184. Doi:10.1111/j.0963-7214.2005.00360.x

Muckli, L., Kohler, A., Kriegeskorte, N., & Singer, W. (2005). Primary visual cortex activity along the apparent-motion trace reflects illusory perception. *PLoS Biology, 3,* e265. Doi:10.1371/journal.pbio.0030265

Öğmen, H., & Herzog, M. H. (2016). A new conceptualization of human visual sensory-memory. *Frontiers in Psychology, 7,* 830. Doi:10.3389/fpsyg.2016.00830

Ruppel, S. E., Fleming, C. N., & Hubbard, T. L. (2009). Representational momentum is not (totally) impervious to error feedback. *Canadian Journal of Experimental Psychology/Revue Canadienne de Psychologie Expérimentale, 63,* 49-58. Doi:10.1037/a0013980

Yantis, S., & Nakama, T. (1998). Visual interactions in the path of apparent motion. *Nature Neuroscience, 1,* 508-512. Doi:10.1038/2226

Supplementary Experiment 3: Dynamic visual noise mask

Supplementary Experiment 3 further tests the claim that predictions of efficient kinematics act on early perceptual representations. It relies on the well-established phenomenon that dynamic visual noise masks, presented directly after stimulus offset, reliably disrupt lower-level perceptual processes (Breitmeyer & Ögmen 2006; Kinsbourne & Warrington, 1962), eliciting similar effects as transcranial magnetic stimulation (TMS) of occipital cortices (for a review, see Tapia & Beck, 2014). Visual masking specifically interrupts re-entrant interactions between V1 and higher visual areas that are crucial for conscious access to a stimulus (Boehler, Schoenfeld, Heinze, & Hopf, 2008; Fahrenfort, Scholte, & Lamme, 2007), either during actual perception (e.g., backwards masking, Lamme, 2000; Lamme & Roelfsema, 2000; Lamme, Zipser & Spekreijse, 2002) or during visual imagery, where masking interferes with the "painting" of top-down information into perceptual structures (e.g., Andrade, Kemps, Werniers, May, & Szmalec, 2002; Borst, Ganis, Thompson & Kosslyn, 2012; McConnell & Quinn, 2000; Quinn and McConnell, 1996, 1999).

To test whether such top-down interactions with early visual processes are responsible for the biases towards efficient actions, we replicated the Report Obstacle experiment but inserted, in half of the trials, a short (560 ms) rapidly changing visual noise pattern immediately after the action offset. Because such dynamic visual noise causes apparent motion (MacKay, 1965), it should interfere with motion based predictions that contribute either to the conscious perception of the seen action, or to the perceptual "filling in" of the suddenly missing information directly after action offset. If the perceptual biases emerge from such changes to early visual perceptual representations, then these biases should be only (or more strongly) observed in the no-mask compared to the masked trials.

Method

Participants

Twenty-eight participants took part in the experiment (mean age = 19.6 years, SD = 1.1, 26 females). All participants were right-handed, had normal/corrected vision, and were recruited from Plymouth University for course credit.

Apparatus & Stimuli

The experiment was presented on a HP EliteDisplay S230tm 23-inch widescreen (1920 X 1080) touch screen monitor. Verbal responses were recorded with Microsoft LifeChat LX-3000 Headsets. All other components of the apparatus were the same as in the main experiments.

The stimulus set was identical to the main experiments. The additional mask stimuli were created in R. The mask covered an area of 200 X 200 pixels and contained 50 black and 50 white squares of equal size (12 X 12 pixels) on a transparent background. Twenty different mask images were created, each containing a randomised arrangement of the squares.

Procedure

The design of the experiment closely matched the Report Obstacle version of the main experiments. Participants completed two blocks of 80 randomised trials. Half of the trials were an exact replication of the Report Obstacle experiment (no-mask condition), and half the trials had the addition of the mask (mask condition), randomly interspersed. Participants again reported whether an obstacle was present in the scene or not, by speaking “Yes” or “No” into the microphone. The action sequence then started and disappeared before completion. In no-mask trials, participants simply indicated on the response stimulus – the scene with the hand removed – the index finger’s last seen location. For masked trials, the

mask was overlaid on top of the response stimulus 560 ms immediately after action offset, on which participants reported – with a touch response – the hand's last seen position. The centre of the mask was positioned at the disappearance point of the tip of the index finger, plus or minus 20 pixels in the X and Y direction, to ensure that participants could not simply use the task to aid their judgment. As soon as the hand disappeared, a sequence of seven randomised mask images was presented at the same rate as the prior action sequence (80 ms per frame), creating a mask which was on screen for 560 ms. Once the mask ended, the response stimulus remained on screen until the touch response was recorded. Any touch responses recorded while the mask remained on screen ended the trial. An example trial sequence for masked trials can be seen in Supplementary Figure 3.

Results

Participant performance and exclusions

Exclusion criteria were identical to the main experiments. No participants were excluded on the basis of the distance between the real and selected screen coordinate (mean = 36.3px, *SD* = 21.9), but one was excluded based on the correlation between the real and selected positions on the X (median r = .944, *SD* = .039) or Y axis (median r = .888, *SD* = .038). A total of 3.2% of trials were excluded due to incorrect response procedure and 2.8% of trials were excluded if initiation or execution times were less than 200 ms or more than 3SD above the sample mean (Initiation: mean = 350.5 ms, *SD* = 158.7; Execution: mean = 527.8 ms, *SD* = 161.8). In 2.9% of trials, a response was made while the mask remained on screen. These trials were included in the analysis but their exclusion/inclusion does not affect the results.

Data analysis

Data was analysed in the same way as the main experiments. Difference values (reported minus actual disappearance points) were entered into a 2 X 2 X 2 repeated-measures ANOVA for the X and Y coordinates separately, with Trajectory (arched, straight), Efficiency (efficient, inefficient), and Condition (mask, no-mask) as within-subjects factors.

Y Axis

Overall, there was a main effect of Trajectory ($F(1,26) = 80.08$, $p < .001$, $\eta_p^2 = .755$) where arched reaches (-1.8px) were displaced higher than straight reaches (-9.7px, $t(26) = 8.74$, $p < .001$, $d = 1.68$). Importantly, the analysis replicated the interaction of Efficiency and Trajectory ($F(1,26) = 22.74$, $p < .001$, $\eta_p^2 = .467$). As before, inefficient arched trajectories (-4.5px) were displaced below efficient arched trajectories (0.7px, $t(26) = -3.71$, $p = .001$, $d = .71$), and inefficient straight trajectories (-7.3px) were displaced above efficient straight trajectories (-12.1px, , $t(26) = 4.25$, $p < .001$, $d = .82$). More importantly, the analysis revealed a three-way interaction between Efficiency, Trajectory and Mask ($F(1,26) = 8.89$, $p = .006$, $\eta_p^2 = .255$). As predicted, the displacements towards the more effective trajectory in the no-mask trials ($F(1,26) = 23.1$, $p < .001$, $\eta_p^2 = .47$) closely resembled the main experiment (14.4 vs. 14.6 pixels, respectively) (Arched/Inefficient vs. Arched/Efficient: $t(26) = -4.06$, $p <.001$, $d = .60$; Straight/Inefficient vs. Straight/Efficient: $t(26) = 3.95$, $p = .001$, $d = .55$). In the masked trials, the Efficiency X Trajectory interaction was substantially reduced (5.2 pixels; ($F(1,26) = 6.8$, $p = .018$, $\eta p^2 = .197$; Arched/Inefficient vs. Arched/Efficient: $t(26) = -1.82$, $p = .081$, $d = .18$; Straight/Inefficient vs. Straight/Efficient: $t(26) = 2.16$, $p = .041$, $d = .28$), Supplementary Figure 3D).

X Axis

Overall, there was a main effect of Trajectory (*F*(1,26) = 148.85, $p < .001$, $\eta_p^2 = .852$). As in the main experiments, arched trajectories (-25.3px) were displaced more leftward than straight trajectories (-4.5px, *t*(26) = -11.98, $p < .001$, $d = 2.3$). An interaction between Trajectory and Mask (*F*(1,26) = 41.41, $p < .001$, $\eta_p^2 = .614$) showed that the Trajectory effect was larger in the no-mask trials than in the masked trials (*t*(26) = 6.44, $p < .001$, $d = 1.2$). There was also a three-way interaction between Efficiency, Trajectory and Mask condition (*F*(1,26) = 4.89, $p = .036$, $\eta_p^2 = .158$) revealing that the Trajectory X Mask condition effect was larger for Efficient actions than for Inefficient actions. While this effect reveals a similar mask effect as for the Y Axis, it should be treated with caution as it was not predicted, no similar interaction of Efficiency and Trajectory was found for any of the main experiments, and it was one of many possible (unpredicted) effects in the ANOVA, and would therefore be subject to adjustments for multiple comparisons (Cramer et al., 2016).

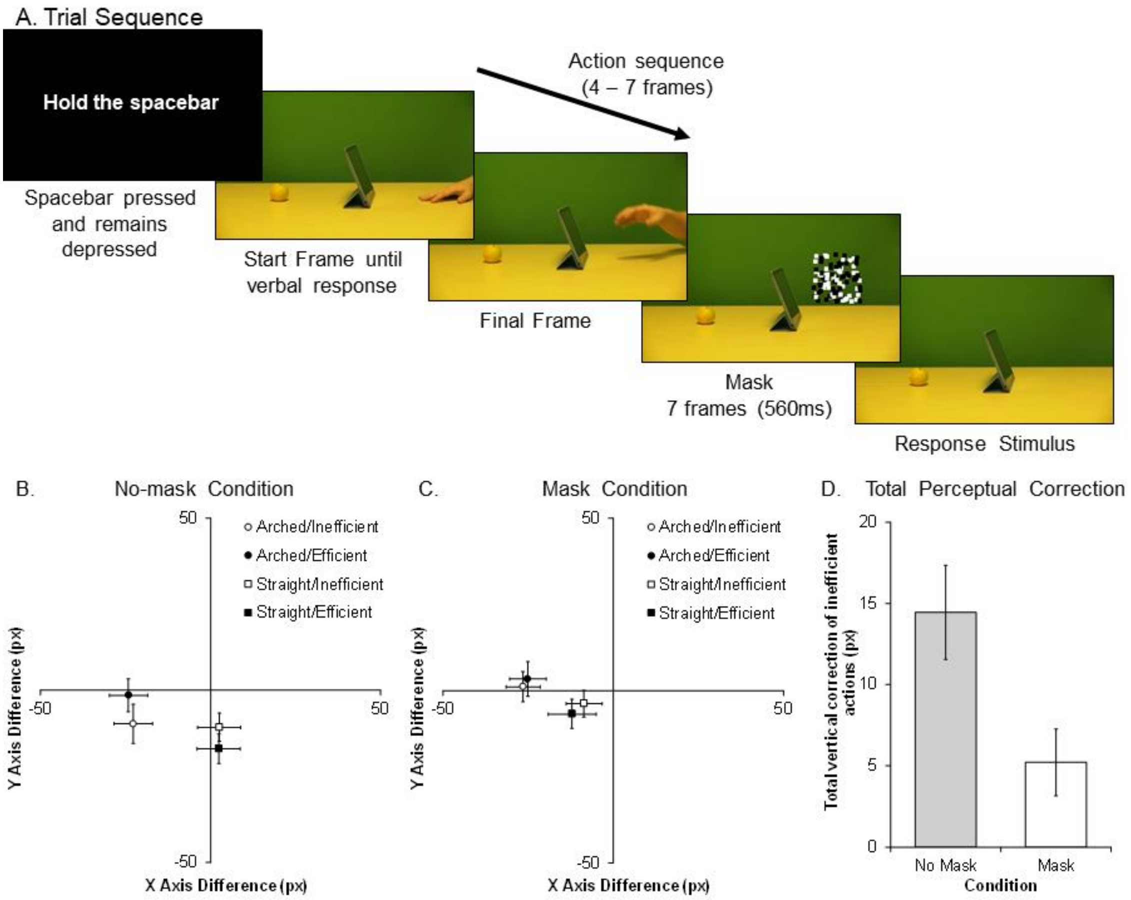

Supplementary Figure 3. Trial sequence and Results for Supplementary Experiment 3. An example of the trial sequence for the Mask condition is depicted in Panel A. The results for the no-mask condition are depicted in Panel B and the results for the Mask condition are depicted in Panel C. Panel D depicts a comparison of the size of the Y axis interaction in pixels, equivalent to the total amount by which inefficient actions were corrected towards a more efficient trajectory. Error bars represent 95% confidence intervals.

Discussion

Supplementary Experiment 3 replicated the finding that perceptual judgments of observed actions are biased towards efficient trajectories. Crucially, it showed that a brief dynamic visual noise mask inserted directly after action offset successfully disrupted the resulting effects on perceptual judgments, substantially reducing the bias towards efficient actions. Dynamic visual noise masks as used here specifically interfere with the re-entrant top-down interactions with early perceptual regions (Boehler et al., 2008; Fahrenfort et al., 2007) that

are crucial for visual awareness of a stimulus (e.g., Lamme & Roelfsema, 2000; Lamme et al., 2002) or the creation of a detailed mental image during visual imagery that is akin to actual perception and which can be accessed for further processing (e.g., Andrade et al., 2002; Borst et al., 2012; McConnell & Quinn, 2000). The masking effects therefore further confirm that the perceptual bias in the main experiments either reflect on-line changes to the action's perceptual representation during observation, or spontaneous "filling in" of the suddenly missing input briefly after its offset, creating an impression of an action displaced towards the anticipated ideal reference trajectory. They cannot be explained in terms of demand characteristics, which were equivalent across both Mask and No-Mask conditions, especially as the two conditions varied rapidly in an unpredictable manner, and participants' attention was equally drawn to the environmental constraints in both conditions.

References

Andrade, J., Kemps, E., Werniers, Y., May, J., & Szmalec, A. (2002). Insensitivity of visual short-term memory to irrelevant visual information. *The Quarterly Journal of Experimental Psychology: Section A, 55,* 753-774. Doi:10.1080/02724980143000541

Boehler, C. N., Schoenfeld, M. A., Heinze, H. J., & Hopf, J. M. (2008). Rapid recurrent processing gates awareness in primary visual cortex. *Proceedings of the National Academy of Sciences, 105,* 8742-8747. Doi:10.1073/pnas.0801999105

Borst, G., Ganis, G., Thompson, W. L., & Kosslyn, S. M. (2012). Representations in mental imagery and working memory: Evidence from different types of visual masks. *Memory & Cognition, 40,* 204-217. Doi:10.3758/s13421-011-0143-7

Breitmeyer, B. & Öğmen, H. Visual masking: time slices through conscious and unconscious vision. Oxford University Press, Oxford, 2006. Doi:10.1093/acprof:oso/9780198530671.001.0001

Cramer, A. O., van Ravenzwaaij, D., Matzke, D., Steingroever, H., Wetzels, R., Grasman, R. P., ... & Wagenmakers, E. J. (2016). Hidden multiplicity in exploratory multiway ANOVA: Prevalence and remedies. *Psychonomic Bulletin & Review, 23,* 640-647. Doi:10.3758/s13423-015-0913-5

Fahrenfort, J. J., Scholte, H. S., & Lamme, V. A. (2007). Masking disrupts reentrant processing in human visual cortex. *Journal of Cognitive Neuroscience, 19,* 1488-1497. Doi:10.1162/jocn.2007.19.9.1488

Kinsbourne, M., & Warrington, E. K. (1962). The effect of an after-coming random pattern on the perception of brief visual stimuli. *The Quarterly Journal of Experimental Psychology, 14,* 223-234. Doi:10.1080/17470216208416540

Lamme, V. A. F. (2000). Neural mechanisms of visual awareness: A linking proposition. *Brain and Mind, 1,* 385–406. Doi:10.1023/A:1011569019782

Lamme, V. A., & Roelfsema, P. R. (2000). The distinct modes of vision offered by feedforward and recurrent processing. *Trends in Neurosciences, 23,* 571-579. Doi:10.1016/S0166-2236(00)01657-X

Lamme, V. A., Zipser, K., & Spekreijse, H. (2002). Masking interrupts figure-ground signals in V1. *Journal of Cognitive Neuroscience, 14,* 1044-1053. Doi:10.1162/089892902320474490

MacKay, D. M. (1965). Visual noise as a tool of research. *The Journal of General Psychology, 72,* 181-197. Doi:10.1080/00221309.1965.9710688

McConnell, J., & Quinn, J. G. (2000). Interference in visual working memory. *The Quarterly Journal of Experimental Psychology: Section A, 53,* 53-67. Doi:10.1080/713755873

Quinn, J. G., & McConnell, J. (1996). Irrelevant pictures in visual working memory. *The Quarterly Journal of Experimental Psychology Section A, 49,* 200-215. Doi:10.1080/713755613

Quinn, J. G., & McConnell, J. (1999). Manipulation of interference in the passive visual store. *European Journal of Cognitive Psychology, 11,* 373-389. Doi:10.1080/713752322

Tapia, E., & Beck, D. M. (2014). Probing feedforward and feedback contributions to awareness with visual masking and transcranial magnetic stimulation. *Frontiers in Psychology, 5,* 1173. Doi:10.3389/fpsyg.2014.01173

Supplementary Table 1

		X Axis				Y Axis			
		Arched		Straight		Arched		Straight	
		Inefficient	Efficient	Inefficient	Efficient	Inefficient	Efficient	Inefficient	Efficient
**Overall**	**Mean**	-24.3	-24.6	7.1	8.3	-19.7	-11.0	-12.0	-17.7
	**SD**	20.5	21.1	18.3	20.3	17.9	19.5	13.2	13.1
	**t(84)**	-10.904	-10.707	3.590	3.758	-10.146	-5.206	-8.355	-12.463
	**p**	0.000	0.000	0.001	0.000	0.000	0.000	0.000	0.000
	**d**	-1.68	-1.61	0.48	0.52	-1.44	-0.66	-1.12	-1.83
	**95% CI (+/-)**	4.4	4.5	3.9	4.3	3.8	4.1	2.8	2.8
**No Task**	**Mean**	-35.1	-35.9	1.2	2.6	-19.3	-14.3	-13.3	-16.0
	**SD**	18.8	19.3	16.6	19.5	15.4	15.0	11.5	11.5
	**t(29)**	-10.225	-10.201	0.408	0.732	-6.830	-5.227	-6.377	-7.635
	**p**	0.000	0.000	0.686	0.470	0.000	0.000	0.000	0.000
	**d**	-2.73	-2.65	0.10	0.17	-1.45	-1.03	-1.44	-1.82
	**95% CI (+/-)**	6.7	6.9	6.0	7.0	5.5	5.4	4.1	4.1
**Report Object**	**Mean**	-11.3	-11.2	15.2	18.0	-21.9	-13.6	-12.7	-18.9
	**SD**	19.2	19.5	21.0	21.4	20.4	21.2	12.3	12.5
	**t(26)**	-3.054	-2.993	3.770	4.366	-5.592	-3.331	-5.389	-7.832
	**p**	0.005	0.006	0.001	0.000	0.000	0.003	0.000	0.000
	**d**	-0.86	-0.78	0.86	1.14	-1.57	-0.81	-1.18	-1.94
	**95% CI (+/-)**	7.2	7.4	7.9	8.1	7.7	8.0	4.6	4.7
**Predict Trajectory**	**Mean**	-25.3	-25.3	5.6	4.9	-18.0	-5.0	-9.9	-18.4
	**SD**	16.7	17.5	14.6	16.8	18.2	21.2	15.9	15.4
	**t(27)**	-8.004	-7.642	2.034	1.540	-5.229	-1.236	-3.288	-6.336
	**p**	0.000	0.000	0.052	0.135	0.000	0.227	0.003	0.000
	**d**	-1.96	-1.90	0.45	0.37	-1.25	-0.26	-0.80	-1.72
	**95% CI (+/-)**	6.2	6.5	5.4	6.2	6.7	7.9	5.9	5.7

Supplementary Table 1. The difference between the real final position and the selected position along the X and Y axis for the efficient and inefficient straight and arched action trajectories, along with the results of a one-sample t-test (test value = 0) for each experiment and overall (*p* values uncorrected).

Supplementary Table 2

		X Axis				Y Axis			
		Arched		Straight		Arched		Straight	
		Inefficient	Efficient	Inefficient	Efficient	Inefficient	Efficient	Inefficient	Efficient
	**Mean**	13.33	13.18	14.33	14.63	-11.63	-9.84	-10.33	-9.78
	**SD**	2.62	4.14	2.96	3.56	3.89	4.61	2.97	4.20
**Supp Exp 1**	**t(13)**	19.03	11.92	18.10	15.40	-11.20	-7.99	-13.04	-8.71
**Non-Biological**	**p**	0.000	0.000	0.000	0.000	0.000	0.000	0.000	0.000
	**d**	2.30	1.73	1.63	1.41	-1.14	-0.73	-1.32	-1.05
	**95% CI (+/-)**	1.37	2.17	1.55	1.86	2.04	2.42	1.55	2.20
	**Mean**	0.06	0.11	-0.03	-0.07	-0.02	0.02	0.00	-0.14
	**SD**	0.16	0.15	0.16	0.14	0.17	0.15	0.15	0.14
**Supp Exp 2**	**t(36)**	2.16	4.52	-1.19	-2.96	-0.90	0.70	-0.15	-6.35
**Probe**	**p**	0.038	0.000	0.242	0.005	0.372	0.490	0.881	0.000
	**d**	0.40	0.90	-0.21	-0.49	-0.18	0.13	-0.02	-1.04
	**95% CI (+/-)**	0.05	0.05	0.05	0.04	0.05	0.05	0.05	0.04
	**Mean**	-22.7	-24.1	2.5	2.4	-9.7	-1.4	-10.8	-16.9
	**SD**	15.2	14.8	16.8	16.8	15.0	12.6	11.0	11.5
**Supp Exp 3**	**t(26)**	-7.77	-8.47	0.78	0.75	-3.36	-0.58	-5.11	-7.67
**No Mask**	**p**	0.000	0.000	0.441	0.460	0.002	0.566	0.000	0.000
	**d**	-1.77	-1.86	0.15	0.14	-0.81	-0.11	-1.04	-1.27
	**95% CI (+/-)**	5.74	5.58	6.34	6.34	5.65	4.75	4.13	4.32
	**Mean**	-26.51	-25.18	-8.61	-12.10	1.13	3.37	-3.79	-6.77
	**SD**	13.39	14.28	17.02	18.65	11.52	13.27	10.32	11.17
**Supp Exp 3**	**t(26)**	-10.28	-9.16	-2.63	-3.37	0.51	1.32	-1.91	-3.15
**Mask**	**p**	0.000	0.000	0.014	0.002	0.614	0.198	0.067	0.004
	**d**	-1.99	-2.23	-0.54	-0.75	0.07	0.31	-0.28	-0.53
	**95% CI (+/-)**	5.05	5.39	6.42	7.04	4.35	5.01	3.89	4.21

Supplementary Table 2. Response biases on the X axis and Y axis for efficient and inefficient arched and straight trajectories in each of the Supplementary Experiments, with the results of a one-sample t-test (test value = 0, *p* values uncorrected).
